# The feasibility of introducing an adult safeguarding measure for inclusion in the Adult Social Care Outcomes Framework (ASCOF): findings from a pilot study

**DOI:** 10.1186/s12913-016-1464-9

**Published:** 2016-06-30

**Authors:** Caroline Norrie, Jill Manthorpe, Cher Cartwright, Pritpal Rayat

**Affiliations:** Social Care Workforce Research Unit (SCWRU), King’s College London, Strand, London, UK; Health and Social Care Information Centre (HSCIC), 4th Floor North, Trevelyan Square, Leeds, UK

**Keywords:** Outcomes, Adult protection, Survey development, Adult safeguarding, Adult social care outcomes framework (ASCOF), Adult abuse

## Abstract

**Background:**

There are currently no national measures in England reporting the experiences of people who have been involved with adult safeguarding services following concerns that they may be at risk of abuse or neglect. The Health and Social Care Information Centre (HSCIC) aimed to develop a new adult safeguarding outcome measure (survey) for local authorities (LAs) that could be added to the Adult Social Care Outcomes Framework (ASCOF). The ASCOF is a national collection of social care outcomes performance indicators collected from the perspective of people receiving partial or total funding from a LA for care services.

**Methods:**

An outcome measure (a face-to-face interview based survey consisting of 7 questions) was piloted in 40 LAs with 382 adults at risk (or their representative) who had been the subject of a safeguarding investigation. The aim was to investigate the feasibility of the survey in three domains: i) if a statistically representative sample of adults at risk (or their family, friend, carer or advocate) could be recruited; ii) analysis of survey responses and its acceptability to participants iii) feedback from LAs about the survey’s administration.

**Results and discussion:**

Overall the survey results met statistical confidence; however the individual results for adults at risk did not, due to the high proportion of representatives who responded because adults at risk were unable. Responses to the survey were generally positive; 72 % of participants felt that the help received during the safeguarding investigation had made them or the adult at risk (if reporting as a proxy) feel ‘*quite a bit’* or ‘*a lot safer’*. These results are the most robust data collected in England on the perspectives of adults at risk and their representatives on safeguarding services. Participants reported they appreciated being asked for feedback. LAs suggested survey administration improvements.

**Conclusions:**

This survey is one way LAs can meet their new legal requirement under the Care Act 2014 to ‘*seek feedback’* from adults at risk about adult safeguarding services. The survey findings provide the first robust evidence that safeguarding services in the main meet their goals of promoting feelings of safety among adults at risk.

**Electronic supplementary material:**

The online version of this article (doi:10.1186/s12913-016-1464-9) contains supplementary material, which is available to authorized users.

## Background

Adult safeguarding is the term given in England to the protection of adults at risk from abuse, mistreatment or neglect. The lead agency undertaking this work is the local authority (LA) that works with a wide group of other organisations including health services and police. Adult safeguarding services in England have been criticised for their limited involvement of adults at risk in the design and evaluation of services [1–3]. There is currently little knowledge about whether adults at risk are satisfied with the support they received during a safeguarding investigation, and therefore a lack of data which can be used to compare outcomes with other LAs, performance manage staff, or inform quality assurance activities. This has implications for benchmarking and resource allocation [2, 4].

Explanations have been offered for the limited involvement of adults at risk in assessing the outcomes from safeguarding investigations. These include ethical and practical reasons arising from the vulnerability or frailty of such individuals and fears that requests for feedback might cause further harm by revisiting times of distress. However, given the general move towards outcome measurement over the last decade, this lack of data is viewed as inconsistent with imperatives to measure service effectiveness in social and health care and both LAs and central government are keen to involve service users in assessing meaningful outcomes.

Mandatory data about adult safeguarding are currently collected by LAs in England (the Safeguarding Adults Returns - SARs), but these are focused on administrative processes (e.g. timescales and categorisation of type of abuse) and numbers (e.g. of referrals or of concluded cases). The effectiveness of SARs (previously the Abuse of Vulnerable Adult (AVA) returns) as a comparative indicator has also been questioned as the thresholds whereby a concern is designated a ‘safeguarding’ case vary across LAs [5, 6]. Such challenges are acknowledged by LAs:

*“Currently Directors and Safeguarding Adults Boards are faced with a plethora of input/output data but no way of telling from it if they really are making any impact. Directors must have a means of knowing what works and how they are making a difference to people.”* [7]

As a result, the Department of Health (DH) and the Association of Directors of Adult Social Services (ADASS), the co-chairs of the Data and Outcomes Board ((DOB) formerly known as the Outcomes and Information Development Board (OIDB)) agreed the development of an adult safeguarding outcome measure which could be considered for inclusion in the Adult Social Care Outcomes Framework (ASCOF) [8]. It recommended that the proposed new measure should be based on a survey of adults at risk who had been through a safeguarding investigation (not those for whom the concern had been addressed without any subsequent investigation). The focus on ‘completed’ investigations reflects case-management work practices in England, where the aim is to resolve and close cases quickly, and new arising concerns with ‘known’ individuals are generally treated as new cases*.* The new outcome measure would be included in Domain 4 of the ASCOF – as Indicator 4C: *proportion of completed safeguarding referrals where people report that they feel safer.*

The ASCOF is a ‘suite’ of online data collection measures produced by the Health and Social Care Information Centre (HSCIC). It presents data on the outcomes and experiences of people on a range of social services by each LA and enables comparisons [8–10]. First introduced in 2011–12, the ASCOF is the responsibility of the DH that sets the content of the Framework through the DOB (this Board consists of sector representatives and DH staff). The HSCIC is responsible for the collection and publication of data to populate these measures but is not responsible for the measures themselves. Currently (2015) two adult safeguarding questions feed into the ASCOF and these are taken from a service user survey which is delivered by post to a sample of all service users in receipt of LA funded social services [11]. These data are not regarded as reliable indicators about standards of adult safeguarding services as the service user survey is not specifically targeted at *adults at risk.* Response rates from adults at risk are therefore reportedly low and it is suspected that responses are under-representative of more vulnerable and incapacitated adults at risk who may be unable to complete and return a survey independently [4].

The need to address what has been termed a ‘*severe lack of* evidence’ [12] about the effectiveness of adult safeguarding has become pressing given the policy and public interest in this subject. This is evident in a plethora of reports and reviews including the review of the multi-agency safeguarding guidance, *No Secrets* [13], the government response to this review [14], initial proposals for legal reform of adult safeguarding [15], the passing of the Mental Capacity Act 2005, which includes measures criminalising ill-treatment and wilful neglect [16], policy to reform adult social care [17] as well as a reports on a series of high profile scandals, such as the Francis Report [18]. The Care Act 2014 [19] placed adult safeguarding on a statutory basis and the Care Act 2014 Statutory Guidance sets out principles for adult safeguarding practice which include suggesting LAs use this survey [19:265]. All documentation relating to conducting this survey is available on the HSCIC website [20].

Concurrently with the development of an adult safeguarding outcome measure, the DH provided financial support to improve outcomes in adult safeguarding under the Making Safeguarding Personal (MSP) sector-led improvement programme. There is synergy between these two initiatives as MSP activity aims to improve adult safeguarding by facilitating a shift in LA emphasis from processes to improving outcomes for people. The aim of MSP is to focus more on developing practitioner understanding of what people wish to achieve, recording their desired outcomes, developing effective responses, and assessing their effectiveness (see [21]). The synergy of these activities means that in some areas the pilot study reported in this current paper was used to underpin MSP local activity.

## Methods

**Fig. 1:**
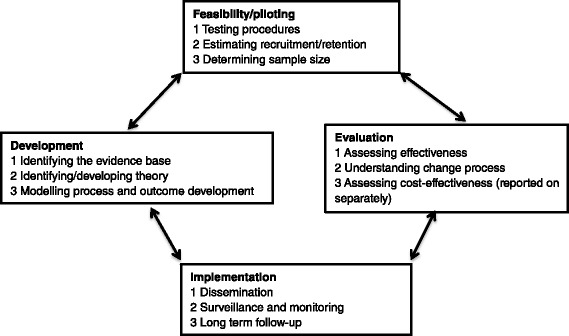
Key elements of the development and evaluation process (Craig et al., 2008, Evans et al., 2013)

This study was informed by an approach outlined in the Medical Research Council’s Guidance on Developing and Evaluating Complex Interventions (MRC GDECI) [22], first published in 2000 [23] and regularly updated [24, 25]. The more recent versions were produced against the background of the Wanless report [26] which problematised the lack of evidence on whether government expenditure achieves policy aims. The MRC GDECI is widely used for carrying out evaluations of complex interventions in healthcare but it has been less frequently applied to social care [27]. Following the MRC GDECI, importance was given to the evaluation cycle which includes:- **Development** (identifying the evidence base; identifying/developing theory; modelling process and outcomes); **Feasibility/piloting** (testing procedures; estimating recruitment/retention; determining sample size); **Evaluation** (assessing effectiveness; understanding change process; assessing cost-effectiveness); **Implementation** (dissemination: surveillance and monitoring; long term follow-up) (see Fig. 1).

Introducing a new adult safeguarding outcome measure meets the definition of a ‘complex intervention’ according to the MRC GDECI [22] as it ‘encompasses a wide range of interacting elements’ (i.e. introduction of the survey in 152 LAs which have different processes in place for organising adult safeguarding involving the NHS, police forces and many other agencies in different ways). ‘Many settings are also involved’ (i.e. individuals’ private dwellings and care homes); there is ‘extensive interplay between different dimensions involved in the delivery and receipt of services’ (i.e. assessments by multi-disciplinary teams working with different user groups accessing a wide range of services or none). The existence of ‘an overriding value base with methodological implications is also relevant (i.e. adults at risk should have equal opportunities to comment on the services they receive, but at the same time they should be protected from further distress and harm); and ‘multiple means are needed to assess the feasibility of the intervention’ (i.e. ascertaining if a representative sample can be recruited, gaining feedback from adults at risk and LA staff, and calculating costs to LAs) [25].

### Developmental work

Following the MRC GDEI [22, 25] focus was placed on developmental work. The pilot study was developed through consultation with the DH, the Association of Directors of Adult Social Services (ADASS), the Personal Social Services Research Unit (PSSRU), Local Government Association (LGA) and lay representatives. It included a HSCIC call for evidence and subsequent analyses of examples of questions posed to adults at risk being used variably by some LAs as part of their own quality assurance processes [21]. Matters discussed at this development stage included processes and outcomes of the survey, i.e. sampling, recruitment, survey questions, inclusion and exclusion criteria, timescales, data analysis, and reporting of findings. Decisions were made by members of a stakeholder group which included the DH and LGA, with representatives from the HSCIC, ADASS, a LA, and a university research team. This group decided that the safeguarding outcomes measure would initially be a national benchmarking measure rather than a local measure as a way of reducing costs. The survey and staff guidance material were developed, then cognitively tested (to ascertain if potential participants understood the material as was intended) in three LAs with 20 adults at risk or their relatives/carers/advocates and LA staff (see [28]) prior to the pilot stage described in this present article. Costs to LAs of introducing the survey were estimated from data collected in the pilot study and are reported on separately [29].

### Feasibility study/piloting

All 152 LAs in England were invited to take part in the eight week pilot study. The aim was for 338 interviews to be undertaken with adults at risk whose ‘case’ had been ‘completed’ following a safeguarding investigation. This number was calculated using the data for the number of completed safeguarding referrals from the Abuse of Vulnerable Adults (AVA) return to HSCIC for 2012/13. It was estimated that for the survey to be undertaken nationally and to be statistically robust (confidence level of 95 %, margin of error 1 %), 8641 interviews would need to be undertaken annually. This would equate to each LA interviewing 10 % of adults at risk or proxies from their total number of completed safeguarding referrals each year.

The pilot started in mid May 2014 and it was planned to run for 8 weeks. The end date was extended by two weeks to accommodate a minority of LAs who requested more time. The 40 participating LAs each agreed to undertake interviews with 20 adults at risk or where these were deemed as not eligible (e.g. lacking capacity, too ill/frail, concerns about further risk identified, had died) interviews would be carried out with those who had supported the adult at risk during the safeguarding investigation or knew the case (relatives, friends, carers, Independent Mental Capacity Advocates (IMCAs)). IMCAs are statutory advocates (under the Mental Capacity Act 2005) who are commissioned by LAs to support and represent people who lack the ability to make important decisions and are appointed where the adult at risk lacks another person to support them in a safeguarding investigation.

Each participating LA was asked to identify safeguarding cases that had closed within the past 8 weeks (on-going throughout the pilot) and assess whether the adult at risk was eligible to take part. LAs were requested to recruit a wide spread of cases and aim to include the following:

- Approximately 3–4 adults at risk from each primary support group using the HSCIC’s classifications (physical, memory and cognition, mental health, learning disability, social support).
- A wide spread across the seven categories of alleged abuse (physical, sexual, psychological/emotional, financial, neglect, discriminatory and institutional).
- Approximately 15 adults at risk with decision making capacity and a further 5 relatives, friends, carers, advocates or IMCAs who had knowledge of the investigation.

LA representatives sent potential participants an information sheet and then telephoned them to ask if they would take part in a face-to-face interview where a survey would be completed (see Additional file 1 for the ‘*Safeguarding Survey for Adults at Risk’* and also the HSCIC website [20]). An additional question (Q8) asked participants for feedback on the survey itself.

LAs were given detailed guidelines about when it would not be appropriate to seek to carry out an interview, whether an interview should be undertaken by two people (e.g. if a risk of violence) and whether an interview should be sought with an adult at risk’s representative (e.g. if an adult at risk was at end of life). The guidance for LAs also contained information on whether to contact potential participants who might be put at increased risk by receiving information about the study, for instance, if they were living with a perpetrator. LAs recruited interviewers from a variety of sources. Where LA staff were used, the guidance stated they should not have been the lead investigator on the cases in question so participants did not feel pressured into answering favourably. Potential interviewees were informed that their responses would be treated confidentially. All participants, who were able, gave written consent to taking part in the pilot and verbal consent was recorded where participants were physically or otherwise unable to sign the form.

LAs were encouraged to contact the HSCIC if they had any questions about administering the survey and a weekly voluntary teleconference meeting was held which acted as a forum for discussing progress and sharing ideas with other LAs.

### Evaluation

The survey process was evaluated to explore: i) whether a statistically representative sample could be collected, ii) the content of the responses to the survey and its acceptability to participants, iii) the nature of LAs’ feedback on conducting the survey.

1. Recruiting a representative sample
  The HSCIC requested details from all LAs about all safeguarding cases that closed between 24th March and 4th July 2014. As noted above, this information is collected routinely by LAs as part of their annual Safeguarding Adults Return (SAR). It includes socio-demographic information about the adult at risk (age group, primary support reason, ethnicity) and about the safeguarding case (type of abuse, source of risk, location of risk, outcome). LAs were then also asked to assess whether each adult at risk or someone who supported them was eligible to take part in the survey, or the reason otherwise, and, if contacted, whether they had agreed or declined to participate.
2. Responses to the survey questions
  The HSCIC asked LAs to provide it with information about all interviews conducted within each LA. This included details about the adult at risk and the safeguarding case (as categorised in the SAR collection), whether the adult at risk was interviewed or someone else, answers to the survey questions (including feedback on the survey itself), the job title of the interviewer, interview length, and date.
3. LA feedback
  LAs were asked to complete an online survey to capture their opinions about the survey and to understand the change process which would be involved in implementing this survey. Opinions were requested on the survey administration guidance documents, participant recruitment, the interview process and development possibilities for the survey. Feedback was also collected through emails and the weekly telephone conference meetings. Cost data are reported separately (see [29]).
  Ethical approvals for the study were obtained in April and May 2015 and included procedures for obtaining written or verbal consent. The study was approved by the Social Care Research Ethics Committee (SCREC) (Ref: 14/IEC08/0016); it was supported by the Association of Directors of Adult Social Services (ADASS) (ref: RG14–007); and research governance approval was also obtained from participating LAs.

## Results

### The starting population

**Fig. 2:**
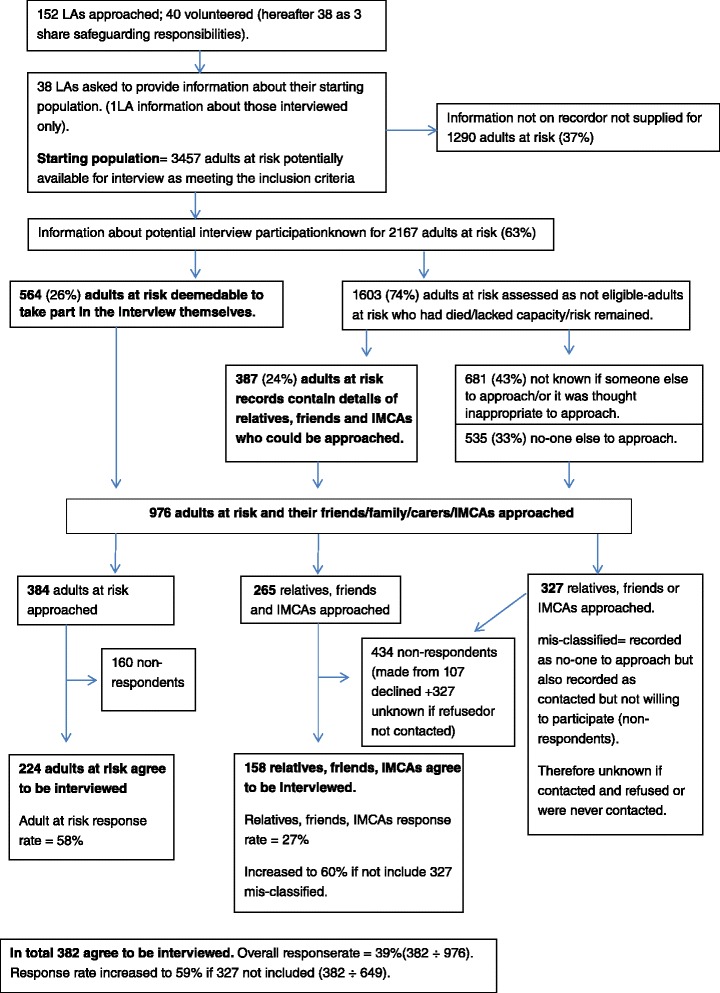
Recruitment Flow Chart

The MRC GDECI guidelines [22] outlines the various steps to piloting/feasibility, including testing procedures, estimating recruitment/retention and determining sample size. Given the vulnerability of the potential participants, it was particularly important to identify the numbers of participants that would need to be recruited if the study was to be adopted nationally and could meet statistical confidence (95 % confidence, 5 % margin of error, following HSCIC methods) (see Fig. 2). In response to the invitation to participate in the pilot sent to 152 LAs, 40 LAs volunteered (including 3 who share safeguarding arrangements and are from here onwards counted as 1 LA thus the number of LAs is henceforth referred to as 38). Out of the 38 LAs, 37 provided information about their starting population other than those interviewed. In total they reported 3457 adults at risk were potentially available for interview (hereafter referred to as the starting population) as meeting the inclusion criteria (case concluded; data collection period). The average number of potential interviewees in each LA was 91 (ranging from 3 to 582 adults at risk per LA in the time period). Not all LAs were able to report further on their starting population and whether they could be approached for an interview as this was a time-consuming task for some LAs with large numbers.

Out of the 3457 adults at risk in the starting population, information about eligibility was initially reported as not known for 1290 individuals (37 %). Information about potential interview participation was known for 2167 adults at risk (63 %). Of the remaining 2167 adults at risk (63 %), 564 (26 %) were deemed eligible to take part in the interview themselves while 1603 adults at risk (74 %) were assessed as not eligible (i.e. lacked decision making capacity (36 %) (for instance, had severe dementia or profound learning disabilities) had died subsequent to the investigation (17 %), or there were concerns around risk (7 %)).

For the 1603 adults at risk identified from the records who were not eligible to take part in the survey themselves, in respect of 387 (24 %) the LA had records of another interested party in the safeguarding investigation who might be approached for interview. For 535 adults at risk (33 %) it was recorded that there was no other person that could be contacted for interview. For 681 (43 %) it was not recorded if someone else available could be contacted for interview or it was felt inappropriate for an interview to be conducted (the case had been closed at the individual’s request or there had been a subsequent safeguarding concern).

In summary, of the 3457 adults at risk in the starting population, 976 adults at risk, or those that supported them, were invited to take part in an interview. Just over a third, 382 (39 %), agreed to take part in the interview while 594 declined (61 %).

Of the 976 individuals who were invited to take part in an interview 384 were adults at risk and 592 were individuals that had supported the adults at risk. Over half of adults at risk (224 of the 384 adults at risk, 58 %) agreed to take part in the survey, whilst only 158 of the 592 individuals that supported adults at risk (27 %) agreed to take part.

It was possible the response rate for individuals that supported adults at risk would have been much higher if it had not been for a mis-categorisation of *non-response* and *ineligible*. LA staff ticked the option, ‘*contacted but declined to take part’* for 434 individuals that supported adults at risk which was noted as ‘***non-respondent***’. However for 327 of these non-respondents, LA staff *also* ticked ‘*the adult at risk ineligible to participate and that there was no other suitable person to interview’* therefore categorising these individuals as ‘***ineligible****’*. The selection of both these categories was contradictory. If those who may have been ‘ineligible’ were removed, the recalculated response rate for individuals that supported adults at risk would be 60 % (158 respondents of 265 contacted) and the overall response rate would be 59 %.

### Responses to the survey questions

Across the 38 LAs participating, as noted, for the survey to meet statistical confidence (95 % confidence, 5 % margin of error) a total of 338 interviews with adults at risk needed to be completed. In total 382 interviews were completed. Overall the survey met statistical confidence; however the individual results for adults at risk did not.

Almost all, 36 of the 38, LAs completed and returned responses for at least one interview whilst the maximum number of interviews per LA was 28. The average number of interviews completed within each LA was 10. Two LAs were not able to complete any interviews within the time-frame (Table 1).

**Table 1:** Interviews conducted by participant groups (total *n* = 382)

Number of interviews conducted by groups		
Individual interviewed	Number of interviews	Percentage of total
Adult at risk	224	58.6
Relative	123	32.2
Advocate (not specified)	3	0.8
Friend	3	0.8
Carer (paid and unpaid)	12	3.1
IMCA	17	4.5

Just over half of the interviews (*n* = 224, 58.6 %) were conducted with adults at risk, with relatives accounting for nearly a third of participants (*n* = 158, 27 %). A small number (17) of interviews were conducted with IMCAs, 6 of which were carried out over the telephone.

The characteristics of those who replied to the survey were compared with the characteristics of the starting population to confirm the survey offered equal opportunities for completion by adults at risk (and their family, friends, carers and IMCAs) across gender and ethnicity. All primary support care ‘client’ groups were represented with physical support accounting for 48 % and learning disability support accounting for 20 % of those interviewed.

Interviews were undertaken with people whose safeguarding cases were classified in one or more of the seven categories of abuse as recorded in the SAR data (physical, sexual, psychological/emotional, financial, neglect and acts of omission, discriminatory, institutional). Participants therefore had agreed to take part even when their cases were potentially highly sensitive, such as sexual abuse where reluctance might have been anticipated. There were however slightly more allegations of financial abuse (30 %) included in the survey population compared to the starting population (21 %) which may reflect greater willingness by this group of participants to take part or that LA staff felt comfortable asking them to participate.

In the majority of the interviews (316) there was no need for additional assistance except for show-cards which had been developed as a visual aid to help participants see all the possible response choices at one time (see [28] and [20]). However, additional assistance was provided in 15 interviews, from an advocate, a communication assistant, or an interpreter.

**Fig. 3:**
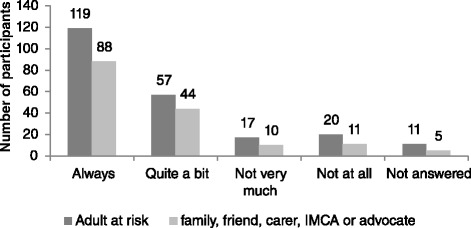
Responses to question 1: “*Did you/the person you support feel listened to during conversations and meetings?”*

**Fig. 4:**
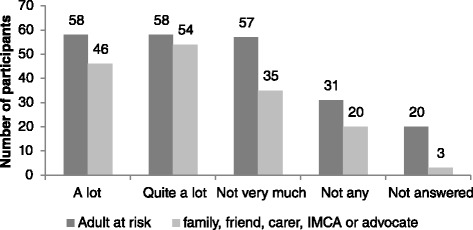
Responses to question 2: *“Did you/the person you support get information during the concern? (This could be spoken or written.)*”

**Fig. 5:**
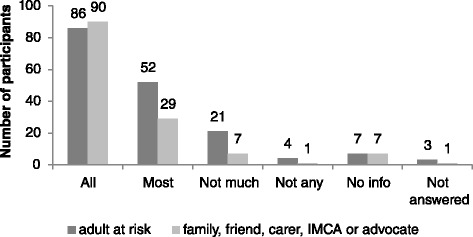
Responses to question 3: “*Were you/the person you support able to understand the information given to you?”*

**Fig. 6:**
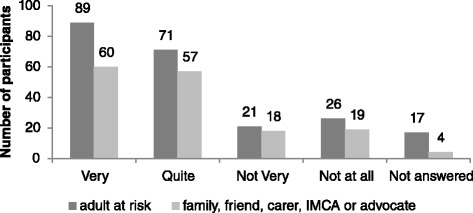
Responses to question 4: *“How happy are you/is the person you support, with the end result?”*

**Fig. 7:**
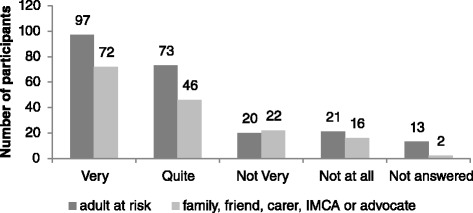
Responses to question 5: *“How happy are you with the way people dealt with your/the person you support’s concern?”*

**Fig. 8:**
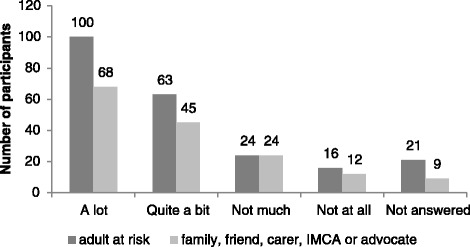
Responses to question 6: *“Do you feel that you are safer now/do you feel that the person you support is safer now because of the help from people dealing with the concern?”*

Survey findings are reported in the following Figs. 3, 4, 5, 6, 7 and 8 demonstrate that responses to most questions were positive. In the majority of cases adults at risk (or their relatives, friends, carers and IMCAs) were happy with the safeguarding service received. Questions 1–5 were used by way of introduction to the topic and were designed to be of use by LAs to gain wider information about views about their services. Question 6 was viewed as the most important measure as it was the potential new ASCOF measure - ***“Do you feel that you are safer now/****do you feel that the person you support is safer now****because of the help from people dealing with the concern?”*** In answer to this question, 72 % of participants felt that the help they had received during the safeguarding investigation had made them feel ‘*a lot safer’ or ‘quite a bit safer’*.

The proportion of participants answering positively was similar for both adults at risk and those that supported them. Adults at risk were less likely to answer individual questions than representatives. Speculatively, this may be because adults at risk could not understand the question or the answer options, or it may indicate reluctance by adults at risk to reflect negatively on the LA that may be providing their support.

It might be expected that that there is a relationship between improving a person’s safety and increasing happiness; however it emerged that safety was not always the only desired outcome. A minority, 12 % of adults at risk and 12 % of relatives, friends, carers and IMCAs, reported they (or the person they supported) were safer, but they were *not* happy with the outcome of the safeguarding investigation. Conversely, 35 % of the adults at risk who reported that they did not feel safer after the safeguarding investigation (*n* = 14) reported they *were* happy with the outcome. Similarly, 39 % of family, friends or carers who stated they did not feel the person they support was safer after the safeguarding investigation, reported being happy with the outcome.

Interviews varied in length from 10 min to two and a half hours, the average length of an interview was 42 min. There was no difference in length of time for interviews conducted with adults at risk or with individuals that supported them. The most frequent time of day for interviews was the afternoon with only six interviews taking place after five pm. A variety of interviewees carried out the survey, ranging from LA safeguarding and performance team staff to student social workers and third sector staff.

As noted in the MRC Guidance [22], testing of procedures being implemented from the perspective of different groups is key to the evaluative process. The perspectives of participants on being involved in this survey were important to capture, given the sensitivity of the survey. Question 8 therefore asked participants *“Is there anything you would like to tell us about the questions or taking part in this interview?*” Comments received were summarised and indicated that adults at risk and their relatives welcomed the opportunity to take part in the survey and to give feedback on services; they found the interview of benefit individually and valued the potential opportunity of helping improve services for others.

### LA staff feedback

The online LA staff feedback survey sought opinions about being involved in the pilot study, including testing of the survey documents, different stages of the pilot (assessing eligibility, recruiting and interviewing) and opinions on the pilot development. Free text boxes were available throughout the feedback survey so detailed information was gained on all aspects involved in testing the survey.

Most (34 out of the 38) LAs participating in the survey submitted answers to most questions. They generally reported the documentation accompanying the survey as useful (telephone scripts for recruitment, information leaflets, consent forms, interview scripts, show cards and help leaflets - all which had been cognitively tested) [28]. Almost all (93 % of LAs) reported holding a centralised list of cases and being able to identify which cases had closed in a previous 8 weeks. About three quarters (72 %) of LAs found collating a list of closed cases very easy or fairly easy, with only one LA reporting it to be ‘very hard’.

However, assessing eligibility and recruiting participants were reported by LAs as time-consuming and resource-intensive. Assessing eligibility of an adult at risk (or a potential relative, friend, carer, advocate or IMCA) was particularly difficult when the case had been closed. Social workers needed to re-familiarise themselves with case records to understand whether a potential participant had capacity to agree to be interviewed or if not whom would be an appropriate alternative, this was time-consuming and often required liaison with colleagues. Processes for identifying potential participants varied across the sites according to how adult safeguarding work was organised and may have been easier where centralised specialist safeguarding teams were present (see [30]). Assessing the risk of contact with potential participants with fluctuating health conditions or changing social circumstances was deemed complex. Half (53 % of LAs) reported this to be *‘fairly hard’* or ‘*very hard’* and none reported it as very easy. Nonetheless, 69 % of LA participants rated the stages of making initial contact and arranging the interview as ‘*moderate,’ ‘fairly easy’* or ‘*very easy’*. The most difficult factor was working within an 8-week time-frame, which 67 % of LAs found *‘fairly hard’* or *‘very hard’*.

Differences between recruiting adults at risk and those that supported them were explored. While not many answered this question, of those that did, most (*n* = 12), reported it had been easier to interview a relative or representative as it was possible to introduce the survey to them by telephone, whereas for adults at risk this was not always appropriate and a direct approach was needed. LA staff commented that ‘cold calling’ potential participants by telephone often required several attempts.

Many LA respondents commented on the sensitivity of contacting adults at risk after their case had closed and the perceived risk of causing emotional distress by reminding them of the event. Some suggested that raising the possibility of seeking feedback through interviews in the communication with safeguarding practitioners during the course of the investigation might be easier and improve recruitment of adults at risk to a later survey. Interestingly, 72 % of LA respondents stated they would ‘*definitely build the survey into their safeguarding’* processes if it were to be a national imperative. Not surprisingly, LAs felt that due to the potentially distressing nature of the interview subject, outcome measures were best elicited face to face with adults at risk (77 %). However, they expressed a preference to conduct some interviews by telephone especially with carers (79) and IMCAs (94 %). Opinion was mixed however on whether it would be appropriate for telephone interviews to be carried out with relatives (not carers) or friends, with 56 % of LAs considering this would be acceptable.

#### Limitations of this study

This pilot study relied on recruiting adults at risk through LAs (following Data Protection Act considerations) with consequent risks of bias. The decision that safeguarding staff or other practitioners would act as ‘gatekeepers’ to participants was also made to minimise potential distress and other risks; the potential that they might be selective is acknowledged but remains. LAs may define ‘completed’ cases differently and this could influence sampling. The extent to which adults at risk (and their relatives, friends, carers, advocates or IMCAs) feel confident that services will not be affected by negative comments about the LA is unknown, although participants did not mention this as a concern. This is the first time data of this nature has been collected therefore there are no previous datasets for comparison.

## Discussion

According to the MRC GDECI [22] evaluations should include assessing the effectiveness of an initiative and also developing understanding of change processes involved. Findings arising from this pilot have been assessed in the light of these comments and the outcome measure is being further scrutinised.

The findings themselves are potentially valuable additions to the evidence on the activity of adult safeguarding, being the most robust data collected in England on the views of adults at risk (and their family, friends, carers and IMCAs) about of the personal experiences of an adult safeguarding investigation. That these survey results are largely positive may reassure adult safeguarding practitioners and managers who are sometimes uncertain about the value of investigations [7].

Whilst the survey was well received by LAs and question 6, which could potentially form the new ASCOF safeguarding outcomes measure, provided useful results to the sector, further work is needed on the development of an effective, worthwhile and usable ASCOF measure for safeguarding. An important matter to resolve, which may be an intrinsic element of this specific measure, is how to analyse, represent and communicate the complex situation where participants who feel themselves (or the adult at risk they support) *safer*, are NOT happy with the outcome of the investigation. Similarly, an adult at risk (or their relative, friend, carer or IMCA) might NOT feel much safer, but still be *happy* with the outcome of the safeguarding investigation. A possible solution to this complication is for two new measures to be introduced, composed of question 5 (satisfaction with how the case was dealt with) *and* question 6 (how safe people now feel) so a more nuanced picture can be presented.

An aim of the pilot was to ascertain if it was feasible for LAs to collect and return the required information to the HSCIC. As noted, although the pilot study met statistical confidence, the individual results for adults at risk did not. Improving recruitment of adults at risk to this survey would therefore be important for any future development and suggestions about an early mention to adults at risk and their representatives of a possible invitation to participate in a feedback survey would need to be explored. Some LAs considered that this approach would ease the burden of assessing eligibility; it would reassure potential participants (eliminating the need for ‘cold calling’); and potentially improve the response rate. It might however raise expectations or fears about how much contact adults at risk were likely to have with professionals and their opportunities for feedback. Further investigation is needed of the availability of assistance such as translation and communication assistance to promote equality of opportunity to take part in such a survey. The pilot highlighted that those people supporting the adult at risk are willing to provide feedback in many instances. Their views of the conduct of the investigation and of the outcome are important, where the adult at risk cannot provide a view.

Previously other measures within ASCOF have included combined measures (from service users and carers e.g. ASCOF measure 11 which captured information from both service users and carers about social contact). However since both these measures are statistically robust on their own; these measures have now been split. Consideration is needed as to how this could be made explicit in ASCOF reporting and communications about safeguarding outcomes. In addition to this there seems to be scope for the introduction of telephone interviews for staff and IMCAs following the result of the pilot. Mixing data sources by combining telephone interviews and face to face interviews in one ASCOF measure again is not usual practice and would demand further statistical exploration.

The Care Act 2014, implemented in April 2015, places new requirements on adult safeguarding services to become Care Act compliant. The Care Act Guidance states that Safeguarding Adult Boards are required to produce an annual report, in which they are asked to consider ‘*what adults who have experienced the process say and the extent to which the outcomes they wanted (their wishes) have been realised*’ [19:265]. The survey is highlighted in the Care Act Guidance [19:265] which suggests that its use ‘*would enable local authorities to better understand the experience of those going through the safeguarding process in their locality and would also facilitate the comparison to other local authorities’*. According to the Guidance, adult safeguarding services should meet certain criteria, including identifying whether adults at risk feel empowered during the investigation/inquiry; whether the adult at risk was involved as much as they wanted to be; whether the investigation was proportionate; and whether there was partnership between people involved in the investigation. The survey captures all these domains in a straightforward way that is potentially accessible for people at risk who may, for example, have learning disabilities or be very frail. The survey questions were developed following cognitive testing which emphasised the necessity of making it as accessible as possible and minimising the distress involved to participants of taking part.

## Conclusions

These are the most robust figures produced on the outcomes of adult safeguarding from the perspective of adults at risk and their representatives. They are likely to be of interest and use to practitioners, policy makers and researchers.

## Abbreviations

ADASS, Data and Outcomes Board (DOB); ASCOF, Adult Social Care Outcomes Framework; Association of Directors of Adult Social Services; AVA, Abuse of Vulnerable Adults; DH, Department of Health; HSCIC, Health and Social Care Information Centre; IMCAs, Independent Mental Capacity Advocates; LA, Local Authority; LGA, Local Government Association; MRC GDECI, Medical Research Council’s Guidance on Developing and Evaluating Complex Interventions; MSP, Making Safeguarding Personal; OIDB, Outcomes and Information Development Board; PSSRU, Personal Social Services Research Unit; SAR, Safeguarding Adult Return

## Additional file

Additional file 1: Adult Social Care Safeguarding Survey Interview Schedule – adult at risk. (DOCX 368 kb)
