# Supplementary material for: The feasibility of introducing an adult safeguarding measure for inclusion in the Adult Social Care Outcomes Framework (ASCOF): findings from a pilot study
Source: BMC Health Serv Res. 2016 Jun 30;16:209. doi: 10.1186/s12913-016-1464-9 (PMC4929787; doi:10.1186/s12913-016-1464-9)
Supplement: Additional file 1: — Adult Social Care Safeguarding Survey Interview Schedule – adult at risk. (DOCX 368 kb) [file 12913_2016_1464_MOESM1_ESM.docx]

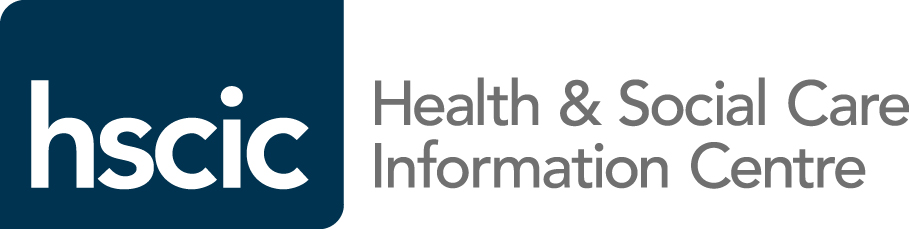


**<<Insert LA logo/name here>>**

**Adult Social Care Safeguarding Survey**

**Interview Schedule – adult at risk**

| **INSTRUCTIONS FOR INTERVIEWER:** *Please clarify the questions to assist the participant if necessary. If the participant would like to see this sheet to help answer the questions, this is fine. Please note, when using the words ‘safe’ and ‘safer’, we are specifically referring to the safeguarding case. When using the word 'people' we are referring to the council and other organisations eg police or home care services.* |
| --- |

**INTERVIEWER READ:**

“*The questions are about your recent experience with the Council when they tried to keep you safe. We will not tell anyone what you say. Your answers will not affect your council services.* **Thank you for your help”.**

LOOKING AT SHOWCARD A
**1**. **Did you feel listened to during conversations and meetings with people about helping you feel safe?**

I was **always** listened to □
I was listened to **quite a bit** □
I was **not** listened to **very much** □
I was **not** listened to **at all** □
Not answered □

LOOKING AT SHOWCARD B
**2. Did you get information during the concern? (This could be spoken or written)**
I got **a lot of** information □
I got **quite a lot** of information □
I did **not get very much** information □
I did **not get any** information □
Not answered □

LOOKING AT SHOWCARD C **3. Were you able to understand the information given to you when people were trying to help you stay safe?**

I was able to understand **all** of the information □
I was able to understand **most** of the information □
I was **not able** to understand **much** of the information □
I was **not able** to understand **any** of the information □
I did **not get any** information □
Not answered □

LOOKING AT SHOWCARD D
**4. How happy are you with the end result of what people did to try and keep you safe?**
I am **very** happy with the end result □
I am **quite** happy with the end result □
I am **not very** happy with the end result □
I am **not at all** happy with the end result □
Not answered □

LOOKING AT SHOWCARD E
**5. How happy are you with how people dealt with the concern throughout?**

I am **very** happy with how people dealt with the concern □
I am **quite** happy with how people dealt with the concern □
I am **not very** happy with how people dealt with the concern □
I am **not at all** happy with how people dealt with the concern □
Not answered □

LOOKING AT SHOWCARD F
**6.** **Do you feel that you are safer now because of the help from people dealing with your concern?**

I feel that I am **a lot** safer now □
I feel that I am **quite a bit** safer now □
I feel that I am **not much** safer now □
I feel that I am **not at all** safer now □
Not answered □

**LOOKING AT SHOWCARD G**

**7a. Is there anything else you think the council (or other
 organisations) could have done better during the time of this
 concern?**

**7b.** **Would you like me to pass on your details so the council
 can contact you further about this?**

Yes □ No, remain anonymous □

About the adult at risk

1. Participant ID number?
2. Does the participant have any special support needs:-

Interpreter (council arranged/informal)

Communication assistance (council arranged/informal)

Other?

1. Is there any other information about the participant which the interviewer should be aware of?

(Where information is missing from council records only the following can be asked at the interview)

1. What is the adult at risk’s gender? Male □ Female □
2. What is the adult at risk’s age? 18-64 □ 65-74 □ 75-84 □ 85-94 □ 95+ □

4. Which of the following ethnic groups listed does the adult at risk
 belong to?

| **A White** |  | **D Black / African /Caribbean/ Black British** |  |
| --- | --- | --- | --- |
| English/Welsh/Scottish/Northern Irish/British Irish Gypsy or Irish traveller Any other white background | □ | African  Caribbean  Other Black background | □  □ □ |
|  | □ |  |  |
|  | □ |  |  |
|  | □ |  |  |
| **B Mixed Multiple ethnic groups** |  | **E Other ethnic group** |  |
| White and Black Caribbean | □ | Arab | □ |
| White and Black African | □ | Other | □ |
| White and Asian | □ |  |  |
| Any other mixed background | □ | **Do not wish to answer** | □ |
| **C Asian / Asian British** |  |  |  |
| Indian | □ |  |  |
| Pakistani | □ |  |  |
| Bangladeshi | □ |  |  |
| Chinese | □ |  |  |
| Other Asian background | □ |  |  |
